# Limitations of VEINES QOL/SYM for discriminating chronic venous insufficiency severity

**DOI:** 10.1590/1677-5449.180096

**Published:** 2019-11-29

**Authors:** Giane Amorim Ribeiro-Samora, Maria Luiza Vieira Carvalho, Regina Márcia Faria de Moura, Danielle A. Gomes Pereira

**Affiliations:** 1 Universidade Federal de Minas Gerais – UFMG, Programa de Pós-graduação em Ciências da Reabilitação, Belo Horizonte, MG, Brasil.; 2 Universidade Federal de Minas Gerais – UFMG, Hospital das Clínicas, Belo Horizonte, MG, Brasil.

**Keywords:** chronic diseases, venous, quality of life

## Abstract

**Background:**

The Venous Insufficiency Epidemiological and Economic Study - Quality of Life/Symptoms (VEINES-QOL / Sym) questionnaire was developed to evaluate the quality of life of individuals with chronic venous insufficiency (CVI), but it has shown limited sensitivity for discriminating between people with different of disease severity.

**Objectives:**

a) to propose a new formula for calculating the VEINES-QOL/Sym score and to evaluate whether this new score is capable of discriminating disease severity; and b) to evaluate the association between VEINES-QOL/Sym scores and disease severity.

**Methods:**

Ninety-eight participants with CVI of both sexes, aged 60.73 ± 14.11 years, answered the Portuguese Brazilian version of the VEINES-QOL/Sym questionnaire. The new score was calculated by transforming the original scores to a 0 to 100 scale. Discriminant analysis was used to test the capability of the original and modified VEINES-QOL/Sym scores to discriminate between and correctly classify groups characterized by the clinical, etiological, anatomical and pathophysiological classification (CEAP). Alpha of 5% was defined as the cutoff for significance.

**Results:**

There were no significant differences between CEAP groups in terms of the original or modified VEINES-QOL/Sym scores. Discriminant analysis was also unable to correctly classify CEAP groups, using either original or modified scores. Furthermore, there were no associations between CEAP classifications and scores obtained using the questionnaire.

**Conclusions:**

The VEINES-QOL/Sym proved to have limitations for assessment of the quality of life and symptomatology of people with CVI at different stages.

## INTRODUCTION

Chronic venous insufficiency (CVI) is a disease involving morphofunctional changes to the venous system, with etiopathogenesis linked to venous hypertension, which in turn may be caused by valve disorders, a thrombotic event, or dysfunction of the triceps surae.^1^

The signs and symptoms of CVI include pain, feelings of heaviness in the lower limbs, burning, cramps, varicose veins, and lipodermatosclerosis, which can compromise a person’s functional capacity.^2^^,^ 3 According to the International Classification of Functioning, Disability and Health model,^4^ it is important to analyze the anatomic and functional changes caused by a disease and to assess the extent to which they affect people’s activity and participation, personal issues, and, consequently, their quality of life.^3^^-^ 5

Chronic venous insufficiency is often assessed using the clinical, etiological, anatomical and pathophysiological classification (CEAP), which characterizes the disease according to its stage of progression.^6^ However, one important aspect, quality of life, is not covered in this classification and is investigated little, despite the fact that an association between CVI severity and worse quality of life is cited in the literature.^7^^-^ 9

Lamping et al.^10^ developed the Venous Insufficiency Epidemiological and Economic Study – Quality of Life/Symptom (VEINES-QOL/Sym) questionnaire to assess the quality of life of people with CVI. This questionnaire has been cross-culturally adapted and validated for the Brazilian population.^11^ It is an instrument with 26 items divided across eight questions that can be used to assess symptoms (VEINES-Sym) and quality of life (VEINES-QOL).

The total score for the VEINES-QOL/Sym questionnaire is based on a standardized score (T score) close to 50,^10^ which can limit the questionnaire’s sensitivity for detecting changes in patients’ quality of life, because the average of the scores for a sample at different stages of disease severity are very similar,^12^ and this is reflected in the questionnaire’s capacity to discriminate between CVI patients in terms of disease severity. Bland et al.^12^ have proposed alternative methods for calculating scores for the VEINES-QOL/Sym that could be capable of detecting longitudinal changes in the quality of life of patients being treated for CVI.

Considering that the signs and symptoms of CVI can have a negative influence on quality of life, that the original method for calculating the VEINES-QOL/Sym score has proved to have low sensitivity for differentiating between people with CVI of different degrees of severity, and that the methods proposed by Bland et al.^12^ for calculating alternative scores are not clearly described, the objectives of this study were: a) to propose a new formula for calculating the VEINES-QOL/Sym score; b) to evaluate whether this new score is capable of discriminating disease severity according to the CEAP classification; and c) to evaluate the association between perceived quality of life as assessed by the modified VEINES-QOL/Sym score and disease severity.

## METHODOLOGY

This study is part of a larger study, approved by the Ethics Committee at the Universidade Federal de Minas Gerais (UFMG), Brazil, with CAAE number 32011414.6.0000.5149. It was conducted with help from Laboratório de Avaliação e Pesquisa em Desempenho Cardiorrespiratório (Labcare), Escola de Educação Física, Fisioterapia e Terapia Ocupacional, Universidade Federal de Minas Gerais (UFMG), and was carried out in the area covered by primary care health center Professor Amílcar Vianna Martins, in the west sanitary region of the Belo Horizonte municipal district. The non-probabilistic sample was selected by searching the Municipal Health Department database. Sample size was calculated on the basis of 20 observations for each independent variable, as proposed by Hair et al.^13^ Participants were enrolled who were over the age of 18, were registered at the primary care health center, had clinical status of CVI, clinically diagnosed by angiologists, and signed free and informed consent forms.

The individuals of the sample were assessed to obtain sociodemographic data and anthropometric data such as weight, height, and body mass index (BMI). A physical examination was conducted to enable analysis of signs and symptoms according to the CEAP classification and the VEINES-QOL/Sym questionnaire was administered by interview. All assessments were conducted by trained examiners.

Participants were classified in relation to CVI severity using the clinical component of the CEAP: C1 = telangiectasias and/or reticular veins; C2 = varicose veins; C3 = venous edema; C4 = skin disorders such as eczema, hyperpigmentation, and lipodermatosclerosis; C5 = healed venous ulcer; C6 = active ulcer.^6^ Both lower limbs were assessed and the classification of the most severely affected limb was used for analysis.

The Brazilian Portuguese version of the VEINES-QOL/Sym questionnaire^11^ was administered in interviews by two trained examiners and the original scores were calculated in accordance with the authors’ instructions.^10^ The VEINES-QOL score was calculated using questions 1, 3, 4, 5, 6, 7, and 8, while for the VEINES-Sym score, questions 1 and 7 were analyzed. Items 3, 6, and 7 were reverse scored. After scoring, means and standard deviations were calculated for each item and then standardization was performed by z score for a mean of zero and standard deviation of one, before calculating the value of each participant’s score minus the mean for the item divided by the standard deviation. The final score was calculated after calculating the mean of all z scores, which were multiplied by 10, and then added to 50. The VEINES-QOL and VEINES/Sym scores were not calculated if fewer than 50% of their respective items had been answered.^11^ Higher scores indicate better outcomes, for both VEINES-QOL and VEINES-Sym.^10^

**Figure 1:**
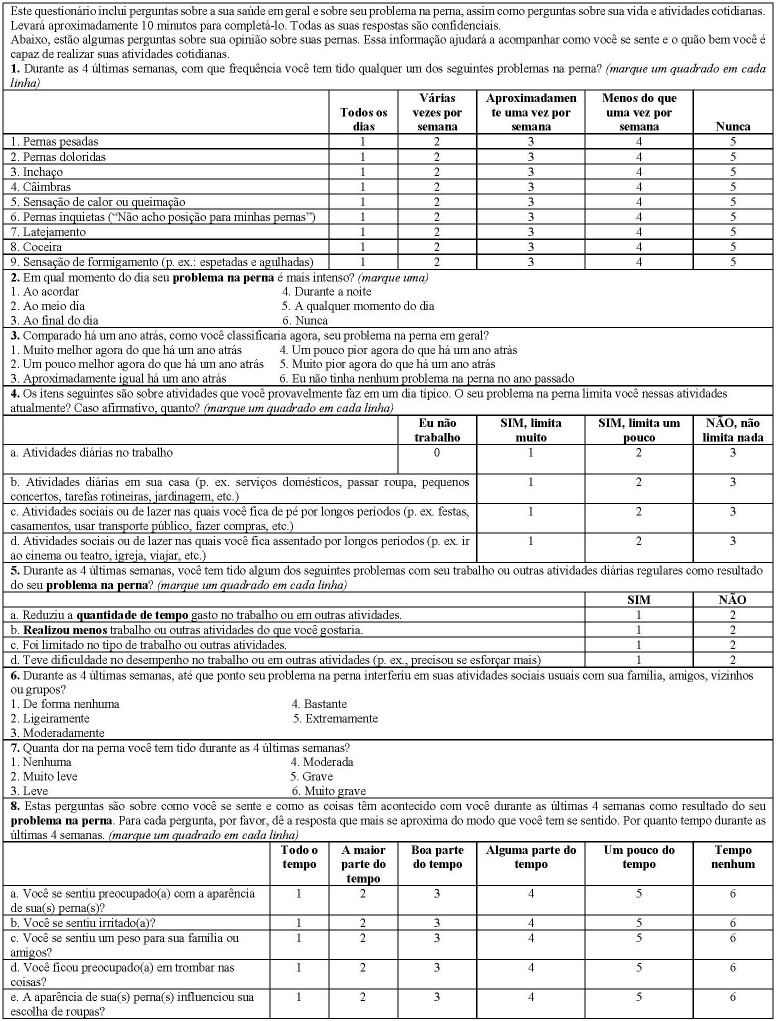
Brazilian Portuguese version of the VEINES-QOL/Sym Questionnaire.

When calculating the new score, both domains, QOL and Sym, were considered, as described in the original article and the values for questions 3, 6, and 7 were inverted as normal. After calculating the VEINES QOL/Sym scores, they were transformed to a scale from 0 to 100, according to the equations shown below, in an attempt to increase the amplitude of the original score. Figure 1 shows the Brazilian Portuguese version of the instrument; see the online appendix to Lamping et al.^10^ for details of the questions and items in the original English version.

To calculate the modified VEINES QOL score, the scores for items 1, 3, 4, 5, 6, 7, and 8 were summed for each volunteer, divided by the sum of the respective maximum score for each item, and then the result was multiplied by 100. This is the equivalent of dividing each volunteer’s score by 1.12 (Equation 1). For the modified VEINES SYM score, the scores for items 1 and 7 were summed for each volunteer, divided by the sum of the respective maximum score for each item, and then the result was multiplied by 100. This is the equivalent of dividing each volunteer’s score by 0.51 (Equation 2).

$$\mathrm{mod}ified\;VEINES\;QOL=\frac{\left(\sum of\;scores\;for\;itens\;1,3,4,5,6,7\;and\;8\right)}{1.12}$$ (1)

$$VEINES\;SYM\;\mathrm{mod}ified=\frac{\left(\sum of\;scores\;for\;itens\;1\;and\;7\right)}{0.51}$$ (2)

### Statistical analysis

Data were expressed as measures of central tendency, distributions, and frequencies. The Shapiro-Wilk test was used to test that the data followed a normal distribution. The sample was divided into two groups according to CEAP disease severity class: a mild CVI group (CEAP 1, 2, and 3) and a group with more advanced stages of the disease (CEAP 4, 5, and 6). Discriminant analysis was used to test the capacity of the original and modified VEINES-QOL/Sym scores to discriminate between and correctly classify the two CEAP groups (CEAP 1, 2, and 3 vs. CEAP 4, 5, and 6), considering a 5% alpha for statistical significance. The Statistical Package for Social Sciences version 15.0 was used for all analyses.

## RESULTS

The sample comprised 98 participants, with a mean age of 60.73 ± 14.11 years, 88.8% (n = 87) female, 24.5% (n = 24) with normal BMI, 31.6% (n = 31) overweight, and 43.9% (n = 43) obese. Arterial hypertension was present in 61% of the sample, diabetes in 25%, and peripheral arterial disease in 1%. Cardiac or respiratory disease was observed in 7%. Disease severity classifications were as follows: CEAP 1, n = 38, CEAP 2, n = 17, CEAP 3, n = 18, CEAP 4, n = 13, CEAP 5, n = 7, and CEAP 6, n = 5.

Mean scores for the entire sample (n = 98) were 52.24 ± 5.22 for the original VEINES QOL; 52.36 ± 6.79 for the original VEINES SYM; 65.81 ± 13.06 for the modified VEINES QOL; and 64.52 ± 13.37 for the modified VEINES SYM. Table 1 shows that there were no differences between CEAP classification groups according to either the original or the modified VEINES-QOL/Sym scores. There were no missing data in the questionnaire responses.

**Table 1:** Characteristics of the sample and comparison of original and modified VEINES scores by CEAP classification groups.

**Variables**	**Mean (95%CI)**		
	**CEAP 1, 2, 3** **(n = 73)**	**CEAP 4, 5, 6** **(n = 25)**	**p**
Original scores			
VEINES_QOL	52.50 (51.28-53.73)	51.48 (49.30-53.67)	0.404
VEINES_SYM	52.52 (50.86-50.86)	51.90 (49.38-54.42)	0.697
Modified scores			
VEINES_QOL	66.28 (63.14-69.42)	64.46 (59.39-69.54)	0.551
VEINES_SYM	65.01 (60.74-69.27)	63.14 (56.87-69.40)	0.646

CEAP = Clinical, Etiological, Anatomic, and Pathophysiological classification; VEINES_QOL = Venous Insufficiency Epidemiological and Economic Study, quality of life domain; VEINES_SYM = Venous Insufficiency Epidemiological and Economic Study, symptoms domain; n = sample size; p = level of statistical significance; 95%CI = 95% confidence interval of mean.

The discriminant analysis could not correctly classify the two CEAP groups (CEAP 1, 2, 3 vs. CEAP 4, 5, 6), whether using the original VEINES-QOL/Sym questionnaire scores or using the modified scores (p > 0.05) (Table 2). Furthermore, there were no associations between CEAP classification and the questionnaire scores (Table 3).

**Table 2:** Results of the discriminant analysis of the original and modified VEINES scores.

**Variables**	**% correct classification of groups**		**% variance**	**Correlation canonical**	**λ Wilks**	**χ^2^_(df = 1)_**	***p*-value**
	**CEAP 123**	**CEAP 456**					
Original scores							
VEINES_QOL	55.6%	52.0%	0.74%	0.086	0.993	0.698	0.404
VEINES_SYM	52.8%	48.0%	0.16%	0.040	0.998	0.151	0.697
Modified scores							
VEINES_QOL	54.2%	44.0%	0.37%	0.061	0.996	0.355	0.551
VEINES_SYM	51.4%	48.0%	0.22%	0.047	0.998	0.211	0.646

CEAP = Clinical, Etiological, Anatomic, and Pathophysiological classification; VEINES_QOL = Venous Insufficiency Epidemiological and Economic Study, quality of life domain; VEINES_SYM = Venous Insufficiency Epidemiological and Economic Study, symptoms domain; λ Wilks = Wilks’s lambda; χ^2^ = chi-square statistic; df = degrees of freedom; p = level of statistical significance.

**Table 3:** Correlations between CEAP classification and original and modified scores for the VEINES-QOL/Sym questionnaire.

**Variables**	**CEAP**	
	**r**	***p*-value**
Original scores		
VEINES_QOL	-0.167	0.103
VEINES_SYM	-0.146	0.154
Modified scores		
VEINES_QOL	-0.109	0.288
VEINES_SYM	-0.149	0.144

CEAP = Clinical, Etiological, Anatomic, and Pathophysiological classification; VEINES_QOL = Venous Insufficiency Epidemiological and Economic Study, quality of life domain; VEINES_SYM = Venous Insufficiency Epidemiological and Economic Study, symptoms domain; r = correlation level; p = level of statistical significance.

## DISCUSSION

The modified VEINES-QOL/Sym score was not capable of discriminating between the different degrees of CVI severity, despite having increased the amplitude compared to the original scores, providing a clearer impression of the measure’s variation. Furthermore, the scores also exhibited no association with disease severity.

Bland et al.,^12^ investigated a sample of patients with venous ulcers and also observed limitations to the scores provided by the VEINES-QOL/Sym, proposing two new methods for obtaining a final score. The first was similar to that used for the SF-36, based on values external to the population; and the second took the values for each question and multiplied them by 100, very similar to what was done in the present study. These calculation methods were compared and correlated together with the original method of calculating the questionnaire scores at three points: baseline, after 2 weeks, and after 4 months. It was observed that the modified scores were able to detect improvements in quality of life and symptomology over time, but the study authors admit that the results may not be the same when analyzed in samples with different degrees of disease severity.^12^^,^ 14 Moura et al.^11^ also found significant differences between groups with CEAP 1, 2, or 3 and CEAP 4, 5, or 6, for the original version of the VEINES-QOL only (p = 0.02), but the correlation analysis showed that there was no association between VEINES/Sym and disease severity.

In the present study, it is possible that the absence of a significant difference between the different CVI stages and absence of an association between quality of life and CEAP classifications are because there really is not association between QoL and disease severity, considering that QoL is multifactorial and aspects such as resilience and coping with the disease cannot be ignored.^15^ Nevertheless, the possibility that the instrument has limited construct validity should also be considered, which is a hypothesis that has already been ventured by Van der Velden et al.,^14^ who observed similar results to those of this study. Although there are studies in the literature that have observed significant associations between CEAP classification and VEINES-QOL/Sym scores,^16^^,^ 17 the associations were of small magnitude, showing that the VEINES-QOL/Sym has limitations that should be better explored.

Quality of life is subjective and multidimensional and is considered an important outcome for assessing the impact that CVI has on people’s lives,^18^ for evaluating the efficacy of treatments,^19^^,^ 20 and for monitoring the progress of their health conditions.^20^

A study by Özdemir et al.,^21^ attempted to evaluate the effect of compression stockings on symptoms and quality of life in 117 patients with CVI at CEAP classes 2 and 3, divided into a control group (exercises) and an intervention group (compression stockings and exercises). This study found that the VEINES score was capable of detecting statistically significant differences after treatment, both in the control group and in the intervention group, but did not detect differences between the groups. This may be related to the similarity of the scores obtained with the questionnaire, which make analysis of the true results difficult.

This study has certain limitations: the majority of the sample was female (88%), which limits the scope for extrapolation of the results to men. On the other hand, it does reflect the real-life situation, since CVI primarily affects women. Another important factor is that the sample of patients classified as CEAP 1-3 was three times larger, i.e. there were many more patients with milder forms of the disease.

## CONCLUSIONS

The inability of both original and modified VEINES-QOL/Sym scores to discriminate CEAP severity and the absence of any association between CEAP classification and the questionnaire scores are evidence that the VEINES-QOL/Sym has limitations with relation to evaluation of the quality of life and symptomology of people with CVI at different stages of severity.
