# Supplementary material for: Limitations of VEINES QOL/SYM for discriminating chronic venous insufficiency severity
Source: J Vasc Bras. 2019 Nov 29;19:e20180096. doi: 10.1590/1677-5449.180096 (PMC6900879; doi:10.1590/1677-5449.180096)
Supplement: Translation

# Translation: Limitação do VEINES QOL/SYM em discriminar a gravidade da insuficiência venosa crônica

**Authors:** Giane Amorim Ribeiro-Samora, Maria Luiza Vieira Carvalho, Regina Márcia Faria de Moura, Danielle A. Gomes Pereira

**Affiliations:** 1 Universidade Federal de Minas Gerais – UFMG, Programa de Pós-graduação em Ciências da Reabilitação, Belo Horizonte, MG, Brasil.; 2 Universidade Federal de Minas Gerais – UFMG, Hospital das Clínicas, Belo Horizonte, MG, Brasil.

**Keywords:** doença crônica, venoso, qualidade de vida

## Abstract

**Contexto:**

O questionário *Venous Insufficiency Epidemiological and Economic Study – Quality of Life/Symptom* (VEINES-QOL/Sym) foi desenvolvido para avaliar a qualidade de vida dos indivíduos com insuficiência venosa crônica (IVC), mas ele tem se mostrado pouco sensível em discriminar indivíduos com diferentes gravidades da doença.

**Objetivos:**

a) Propor uma nova forma de cálculo do escore do VEINES-QOL/Sym e avaliar se esse novo escore é capaz de discriminar a gravidade da doença; b) Avaliar a associação entre o VEINES-QOL/Sym e a gravidade da doença.

**Métodos:**

Noventa e oito participantes com IVC, de ambos os sexos, idade 60,73 ± 14,11 anos, responderam o questionário VEINES-QOL/Sym, versão português do Brasil. Para o cálculo do novo escore, foi realizada uma transformação dos escores originais para uma escala de 0 a 100. Para verificar a capacidade dos escores do VEINES-QOL/Sym, originais e modificados, em discriminar e classificar corretamente os grupos caracterizados pela classificação clínica, etiológica, anatômica e patofisiológica (CEAP), foi realizada a análise discriminante, sendo considerado significante um alfa de 5%.

**Resultados:**

Não houve diferença significativa entre os grupos classificados pela CEAP quanto aos escores originais e modificados do VEINES-QOL/Sym. A análise discriminante também não foi capaz de classificar corretamente os grupos CEAP, tanto para os escores originais quanto para os escores modificados do VEINES-QOL/Sym. Além disso, não houve associação entre a classificação CEAP e os escores obtidos pelo questionário.

**Conclusões:**

O VEINES-QOL/Sym mostrou-se limitado para avaliar qualidade de vida e sintomatologia dos indivíduos com diferentes estágios de IVC.

## INTRODUÇÃO

A insuficiência venosa crônica (IVC) é uma doença com alterações morfofuncionais no sistema venoso, sendo a etiopatogenia associada à hipertensão venosa que pode ser causada por uma modificação valvar, um evento trombótico ou uma disfunção do tríceps sural^1^.

A IVC tem como sinais e sintomas dor, sensação de peso em membros inferiores, queimação, cãibras, varizes e lipodermatoesclerose, que podem levar à redução da capacidade funcional do indivíduo^2^^,^ 3. De acordo com o modelo da Classificação Internacional de Funcionalidade, Incapacidade e Saúde^4^, é importante a análise das alterações anatômicas e funcionais resultantes da instalação da doença, assim como avaliar de que forma essas modificações afetam o nível de atividade e participação dos indivíduos, as questões pessoais e, consequentemente, a qualidade de vida^3^^-^ 5.

A avaliação da IVC é frequentemente realizada por meio da Classificação Clínica, Etiológica Anatômica e Patológica (CEAP), que permite caracterizar o quadro da doença de acordo com sua progressão^6^. Porém, um aspecto importante, como qualidade de vida, não é abordado nessa classificação e é pouco investigado, apesar de, historicamente na literatura, ser citada a associação entre a gravidade da IVC e a piora da qualidade de vida^7^^-^ 9.

Lamping et al.^10^ desenvolveram o questionário *Venous Insufficiency Epidemiological and Economic Study – Quality of Life/Symptom* (VEINES-QOL/Sym) com objetivo de avaliar a qualidade de vida dos indivíduos com IVC. Esse questionário, validado e adaptado transculturalmente para a população brasileira^11^, consiste em um instrumento com 26 itens e oito questões que permitem avaliar os sintomas (VEINES-Sym) e a qualidade de vida (VEINES-QOL).

O escore total do questionário VEINES-QOL/Sym é baseado em um escore padronizado (escore T) que é próximo a 50^10^, o que pode limitar a sensibilidade do questionário em detectar mudanças na qualidade de vida dos indivíduos, uma vez que a média do escores de uma amostra, em diferentes estados de gravidade da doença, são muito semelhantes^12^, o que refletiria na capacidade do questionário em discriminar indivíduos com IVC quanto à gravidade da doença. Bland et al.^12^ propuseram outras formas de calcular os escores do VEINES-QOL/Sym que fossem capazes de detectar alterações longitudinais na qualidade de vida de pacientes em tratamento de IVC.

Considerando que os sinais e sintomas da IVC podem ter influência negativa na qualidade de vida, que a atual forma de cálculo do escore do VEINES-QOL/Sym até o momento tem se mostrado pouco sensível em discriminar indivíduos com IVC de diferentes gravidades e que os métodos propostos por Bland et al.^12^ para calcular novos escores não são descritos claramente, os objetivos deste estudo foram: a) propor uma nova forma de cálculo do escore do questionário VEINES-QOL/Sym; b) avaliar se o novo escore é capaz de discriminar a gravidade da doença, dada pela classificação CEAP; c) avaliar a associação entre a percepção de qualidade de vida obtida pelo escore modificado do VEINES-QOL/Sym com a gravidade da doença.

## METODOLOGIA

Este artigo é parte de um estudo maior, aprovado pelo comitê de ética da Universidade Federal de Minas Gerais (UFMG) sob o parecer CAAE 32011414.6.0000.5149. Ele contou com o apoio do Laboratório de Avaliação e Pesquisa em Desempenho Cardiorrespiratório (Labcare), da Escola de Educação Física, Fisioterapia e Terapia Ocupacional da Universidade Federal de Minas Gerais (UFMG), e foi realizado na área de abrangência da unidade básica de saúde (UBS) Professor Amílcar Vianna Martins, localizada no distrito Sanitário Oeste no município de Belo Horizonte. A amostra não probabilística foi realizada por meio de busca no programa Gestão do Sistema de Informação Eletrônico da Secretaria Municipal de Saúde. O cálculo amostral baseou-se no princípio de 20 observações para cada variável independente, conforme proposto por Hair et al.^13^. Foram incluídos participantes maiores de 18 anos, cadastrados na UBS, com quadro clínico de IVC, clinicamente diagnosticados por angiologistas e que assinaram o termo de consentimento livre esclarecido.

A avaliação da amostra foi realizada para a obtenção de dados sociodemográficos e antropométricos como peso, estatura e índice de massa corporal (IMC). Posteriormente, foi realizado o exame físico para a análise dos sinais e sintomas de acordo com a CEAP e a aplicação do questionário VEINES-QOL/Sym por meio de entrevista. Todas as avaliações foram realizadas por examinadores previamente treinados.

Os participantes foram classificados em relação à gravidade da IVC pela parte clínica da CEAP: C1 = presença de teleangiectasias e/ou veias reticulares; C2 = presença de veias varicosas; C3 = presença de edema venoso; C4 = alterações de pele como eczema, hiperpigmentação, lipodermatoesclerose; C5 = úlcera venosa cicatrizada; C6 = presença de úlcera ativa^6^. Os dois membros inferiores foram avaliados e foi considerada a classificação do membro que se apresentava mais afetado.

O questionário VEINES-QOL/Sym, versão português do Brasil^11^, foi aplicado por meio de entrevista por dois examinadores treinados, e os escores originais foram calculados de acordo com as instruções dos autores^10^. Para o cálculo do escore VEINES-QOL, foram utilizadas as questões 1, 3, 4, 5, 6, 7 e 8, e, para obtenção do VEINES-Sym, as questões de 1 e 7 foram analisadas. Foi realizada a inversão das questões 3, 6 e 7 para, posteriormente, obterem-se a média e o desvio padrão de cada item, com posterior padronização em um escore Z de média zero e desvio padrão igual a um, e cálculo do valor do escore do indivíduo menos a média da questão dividida pelo desvio padrão. O escore final foi obtido após cálculo da média de todos os escores Z, multiplicando-os por 10 e somando 50. Os escores VEINES-QOL e VEINES/Sym não foram calculados se menos de 50% dos seus respectivos itens não tiverem sido respondidos^11^. Escores maiores indicam melhores desfechos para o VEINES-QOL e para o VEINES-Sym^10^.

Para o cálculo do novo escore, foram levados em consideração os dois domínios QOL e Sym, conforme descrito no artigo original, como também a inversão dos valores das questões 3, 6 e 7. Após a obtenção da pontuação no VEINES QOL/Sym, foi realizada a transformação para uma escala de 0 a 100, de acordo com as equações abaixo, a fim de tentar aumentar a amplitude do escore original (Figura 1).

**Figura 1:** Questionário VEINES-QOL/Sym versão português do Brasil.

Para o escore VEINES QOL modificado, foi realizada a soma da pontuação dos itens 1, 3, 4, 5, 6, 7 e 8 de cada voluntário, dividida pela soma da pontuação máxima desses respectivos itens e o resultado multiplicado por 100. Isso equivale a dividir a pontuação obtida pelo voluntário por 1,12 (Equação 1). Para o escore VEINES SYM modificado, foi realizada a soma da pontuação dos itens 1 e 7 de cada voluntário, dividida pela soma da pontuação máxima desses respectivos itens e o resultado multiplicado por 100. Isso equivale a dividir a pontuação obtida pelo voluntário por 0,51 (Equação 2).

$$VEINES\;QOL\;\mathrm{mod}ificado=\frac{\left(\sum da\;pontuação\;nos\;itens\;1,3,4,5,6,7\;e\;8\right)}{1,12}$$ (1)

$$VEINES\;SYM\;\mathrm{mod}ificado=\frac{\left(\sum da\;pontuação\;nos\;itens\;1\;e\;7\right)}{0,51}$$ (2)

### Análise estatística

Os dados foram apresentados por meio de medidas de tendência central, dispersão e frequência. A distribuição normal dos dados foi verificada por meio do teste de Shapiro-Wilk. A amostra foi dividida em dois grupos de acordo com a gravidade da doença pela CEAP: grupo de IVC leve (CEAP 1, 2, 3) e grupo com estágios mais avançados da doença (CEAP 4, 5, 6). Para verificar a capacidade dos escores do VEINES-QOL/Sym, originais e modificados, em discriminar e classificar corretamente os dois grupos CEAP (CEAP 1, 2, 3 *versus* CEAP 4, 5, 6), foi realizada a análise discriminante, sendo considerado um alfa de 5% para significância estatística. Para todas as análises, foi utilizado o *software Statistical Package for Social Sciences*, versão 15.0.

## RESULTADOS

A amostra foi constituída de 98 participantes, com média de idade de 60,73 ± 14,11 anos, sendo que 88,8% (n = 87) eram do sexo feminino, 24,5% (n = 24) apresentavam IMC normal, 31,6% (n = 31) apresentavam sobrepeso e 43,9% (n = 43) eram obesos. Hipertensão arterial estava presente em 61% da amostra, diabetes em 25% e doença arterial periférica em 1%. A presença de doença cardíaca ou respiratória foi observada em 7%. Em relação à classificação: CEAP 1 = 38 indivíduos, CEAP 2 = 17, CEAP 3 = 18, CEAP 4 = 13, CEAP 5 = 7, CEAP 6 = 5.

Os escores médios da amostra geral (n = 98) foram de 52,24 ± 5,22 para o VEINES QOL original, de 52,36 ± 6,79 para o VEINES SYM original, 65,81 ± 13,06 VEINES QOL modificado e de 64,52 ± 13,37 para o VEINES SYM modificado. A Tabela 1 mostrou que não houve diferença entre os grupos classificados pela CEAP quanto aos escores originais e modificados do VEINES-QOL/Sym. Não houve perda de dados nas respostas do questionário.

**Tabela 1:** Caracterização da amostra e comparação dos escores VEINES originais e modificados por grupos, conforme a classificação CEAP.

**Variáveis**	**Média (IC95%)**		
	**CEAP 1, 2, 3** **(n = 73)**	**CEAP 4, 5, 6** **(n = 25)**	**p**
Escores originais			
VEINES_QOL	52,50 (51,28-53,73)	51,48 (49,30-53,67)	0,404
VEINES_SYM	52,52 (50,86-50,86)	51,90 (49,38-54,42)	0,697
Escores modificados			
VEINES_QOL	66,28 (63,14-69,42)	64,46 (59,39-69,54)	0,551
VEINES_SYM	65,01 (60,74-69,27)	63,14 (56,87-69,40)	0,646

CEAP = Classificação Clínica, Etiológica Anatômica e Patológica; VEINES_QOL = *Venous Insufficiency Epidemiological and Economic Study*, domínio de qualidade de vida; VEINES_SYM = *Venous Insufficiency Epidemiological and Economic Study*, domínio referente aos sintomas; n = tamanho da amostra; p = nível de significância estatística; IC95% = intervalo de confiança de 95% da média.

A análise discriminante não foi capaz de classificar corretamente os dois grupos CEAP (CEAP 1, 2, 3 *versus* CEAP 4, 5, 6), tanto para os escores originais quanto para os escores modificados do questionário VEINES-QOL/Sym (p > 0,05) (Tabela 2). Além disso, não houve associação entre a classificação CEAP e os escores obtidos pelo questionário (Tabela 3).

**Tabela 2:** Resultados da análise discriminante dos escores originais e modificados do VEINES.

**Variáveis**	**% classificação correta dos grupos**		**% variância**	**Correlação canônica**	**λ Wilks**	**χ^2^_(gl = 1)_**	**p-valor**
	**CEAP 123**	**CEAP 456**					
Escores originais							
VEINES_QOL	55,6%	52,0%	0,74%	0,086	0,993	0,698	0,404
VEINES_SYM	52,8%	48,0%	0,16%	0,040	0,998	0,151	0,697
Escores modificados							
VEINES_QOL	54,2%	44,0%	0,37%	0,061	0,996	0,355	0,551
VEINES_SYM	51,4%	48,0%	0,22%	0,047	0,998	0,211	0,646

CEAP = Classificação Clínica, Etiológica Anatômica e Patológica; VEINES_QOL = *Venous Insufficiency Epidemiological and Economic Study*, domínio de qualidade de vida; VEINES_SYM = *Venous Insufficiency Epidemiological and Economic Study*, domínio referente aos sintomas; λ Wilks = lambda de Wilks; χ^2^ = quiquadrado; gl = graus de liberdade; p = nível de significância estatística.

**Tabela 3:** Correlação entre a classificação CEAP e os escores originais e modificados do questionário VEINES-QOL/Sym.

**Variáveis**	**CEAP**	
	**r**	**p-valor**
Escores originais		
VEINES_QOL	-0,167	0,103
VEINES_SYM	-0,146	0,154
Escores modificados		
VEINES_QOL	-0,109	0,288
VEINES_SYM	-0,149	0,144

CEAP = Classificação Clínica, Etiológica Anatômica e Patológica; VEINES_QOL = *Venous Insufficiency Epidemiological and Economic Study*, domínio de qualidade de vida; VEINES_SYM = *Venous Insufficiency Epidemiological and Economic Study*, domínio referente aos sintomas; r = nível de correlação estatística; p = nível de significância estatística.

## DISCUSSÃO

O escore modificado do VEINES-QOL/Sym não foi capaz de discriminar as diferentes gravidades da IVC, apesar de aumentar a amplitude dos escores originais e possibilitar uma melhor visualização em relação à variação dessa medida. Adicionalmente, os escores também não mostraram associação com a gravidade da doença.

Bland et al.^12^, em uma amostra constituída de indivíduos que apresentavam úlceras venosas, também observaram as limitações nos escores obtidos pelo VEINES-QOL/Sym e propuseram duas novas formas de se obter o escore final. A primeira era similar ao SF-36, tendo como base valores externos da população; e a segunda forma considerava os valores de cada questão e multiplicava por 100, muito similar ao realizado pelo presente estudo. Essas formas de cálculo foram comparadas e correlacionadas conjuntamente com a forma original de calcular o questionário em três momentos, inicial, após 2 semanas e após 4 meses. Foi possível observar que os escores modificados foram capazes detectar melhora na qualidade de vida e na sintomatologia ao longo do tempo, mas o próprio autor admite que esses resultados talvez não sejam semelhantes quando analisados em amostras com diferentes gravidades da doença^12^^,^ 14. Moura et al.^11^ também encontraram diferença significativa entre os grupos CEAP 1, 2 e 3 e CEAP 4, 5 e 6 apenas para o escore do VEINES-QOL, versão original (p = 0,02), mas a análise de correlação mostrou não haver associação entre o VEINES/Sym e a gravidade da doença.

No presente estudo, é possível que a ausência de diferença significativa entre os diferentes estágios da IVC e a ausência de associação entre qualidade de vida e a classificação CEAP sejam resultantes da real falta de associação entre QV e gravidade da doença, considerando que QV é multifatorial e que os aspectos como resiliência e enfrentamento perante à doença não podem ser negligenciados^15^. No entanto, uma limitada validade de construto do instrumento também dever ser considerada, hipótese essa já levantada por Van der Velden et al.^14^, que encontrou resultados semelhantes ao deste estudo. Embora haja, na literatura, estudos^16^^,^ 17 que observaram associação significativa entre a classificação CEAP e os escores do VEINES-QOL/Sym, a associação foi de baixa magnitude, mostrando que o VEINES-QOL/Sym possui limitações que precisam ser mais bem exploradas.

A qualidade de vida é subjetiva e multidimensional, sendo considerada um desfecho importante para verificar o impacto da IVC na vida dos indivíduos^18^, para avaliar eficácia de tratamentos^19^^,^ 20 e acompanhar a progressão da condição de saúde^20^.

No estudo de Özdemir et al.^21^, foi proposto avaliar o efeito da meia compressiva nos sintomas e na qualidade de vida em 117 pacientes com IVC com CEAP de 2 e 3, divididos em grupo controle (exercícios) e intervenção (uso da meia compressiva e exercícios). Neste estudo, foi possível observar que o VEINS foi capaz de detectar diferença estatisticamente significativa após tratamento, tanto no grupo controle quanto no grupo intervenção, porém não foi encontrada diferença entre os grupos. Esses achados podem estar associados aos escores similares que são obtidos pelo questionário, os quais dificultam a análise de resultados reais.

Este estudo apresenta algumas limitações: a amostra foi constituída, em sua maioria, por mulheres (88%), o que torna a extrapolação dos resultados limitada para os homens. Por outro lado, também reflete a realidade da IVC que acomete principalmente as mulheres. Outro fator importante é o fato de a amostra ser três vezes maior nos indivíduos classificados como CEAP 1-3, ou seja, a forma mais branda da doença.

## CONCLUSÃO

A incapacidade dos escores originais e modificados do VEINES-QOL/Sym em discriminar a gravidade CEAP e ausência de associação entre a classificação CEAP e os escores do questionário evidenciou as limitações VEINES-QOL/Sym em relação à avaliação da qualidade de vida e da sintomatologia dos indivíduos com diferentes estágios de IVC.
